# A protocol to transform a fluorescent reporter from a nuclear to a cytoplasmic location.

**DOI:** 10.17912/micropub.biology.000954

**Published:** 2024-01-17

**Authors:** G. Robert Aguilar, Oliver Hobert

**Affiliations:** 1 Columbia University, New York, New York, United States

## Abstract

To facilitate cell identification for expression pattern analysis in *C. elegans* , an SL2::GFP::H2B fluorescent reporter cassette has become a popular and widely used choice to generate nuclear localized reporter alleles by CRISPR/Cas9 genome engineering. When added at the 3’ end of a locus of interest, this cassette concentrates GFP into the nucleus and permits the identification of expressing cells, for example with the help of the NeuroPAL tool. However, there are instances in which it is desirable to visualize the complete morphology of a cell that expresses an SL2::GFP::H2B reporter cassette. We describe here a CRISPR/Cas9-engineering strategy to transform an endogenous SL2::GFP::H2B tag into a cytosolic tag by insertion of the self-cleaving T2A tag in between GFP and H2B.

**Figure 1. An endogenous nuclear localized SL2::GFP::H2B reporter can be converted to label the cytoplasm by insertion of a T2A sequence:**
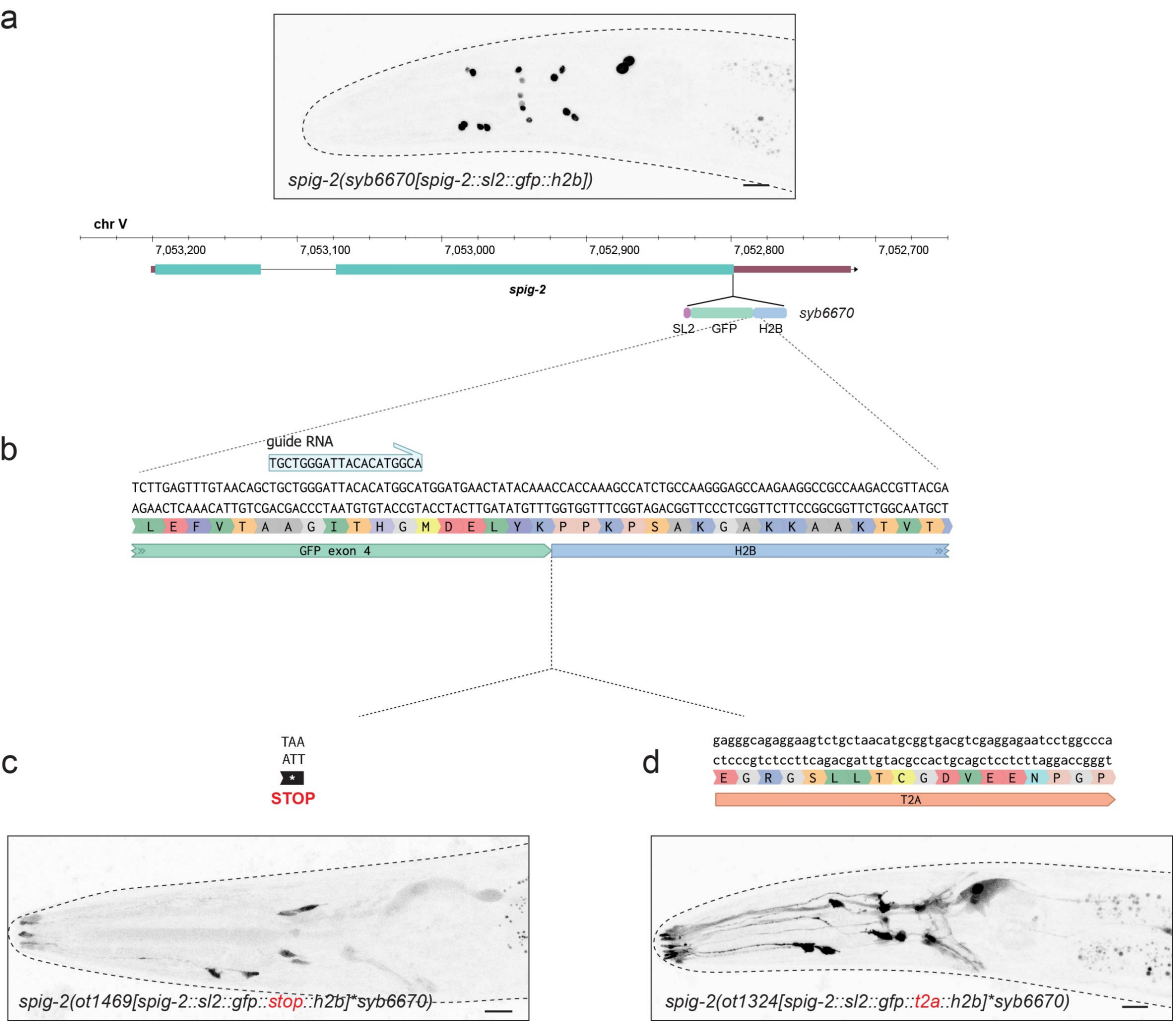
(a) The endogenous locus of *spig-2* (aka *txt-17* ) was tagged with an SL2::GFP::H2B cassette to label the nuclei of cells expressing this gene. (b) To transform this nuclear-localized reporter into a cytoplasmic one, we used CRISPR/Cas9 genome engineering to cut into the last exon of *gfp* using the guide RNA shown. (c) Insertion of a STOP codon between GFP and H2B leads to incomplete conversion of the signal from nuclear to cytoplasmic, but the intensity of the GFP is greatly reduced and variable from animal to animal. (d) Insertion of a T2A sequence between GFP and H2B releases GFP into the cytoplasm, labeling the entire cell. The resulting cytoplasmic signal is robust and consistent across animals. The retention of some GFP in the nucleus suggests that the cleavage efficiency of T2A is less than 100%. Scale bars represent 10 μm. The complete sequence of the guide RNA and repair templates can be found in the Reagents section.

## Description

Gene expression pattern analysis in the compact nervous system of the nematode *C. elegans* is facilitated by the use of nuclear localized reporter approaches. Nuclear-localized fluorophore signals in compact ganglia are discrete and easily separable from nuclear signals from other neurons. Moreover, the identity of expressing neurons can be determined with the NeuroPAL transgene, a multicolor cell identification tool that overlays nuclear GFP signals with neuron-type specific multicolor fluorophore signals (Yemini *et al.* 2021). Also, nuclear localized reporters concentrate fluorescent proteins into the nucleus, thereby providing a stronger and more easily quantifiable signal than a fluorophore that diffuses through the entire cell. The *C. elegans* community has generated scores of reporter alleles over the years in which an SL2::GFP::H2B cassette has been inserted at the 3’ end of genes to identify their sites of expression in the nervous system [e.g. (Fry *et al.* 2021; Sun and Hobert 2021; Cros and Hobert 2022; Vidal *et al.* 2022; Boeglin *et al.* 2023; Brocal-Ruiz *et al.* 2023; Ripoll-Sanchez *et al.* 2023; Sun *et al.* 2023; Yu *et al.* 2023)]. The SL2 trans-splice leader sequence is usually inserted if the expression of a non-nuclear localized protein is examined. In the example shown in Figure 1a, we used CRISPR /Cas9 genome engineering to tag a locus *, spig-2 (* aka *txt-17 )* , that codes for a small secreted peptide, with the SL2::GFP::H2B cassette. This reporter allele allows us to visualize *spig-2* gene expression in multiple glial cells ( Figure 1a ). The nature of the gene and exact sites of expression will be described elsewhere. Apart from identifying the sites of expression of the *spig-2* gene, this reporter allele is a valuable cell fate reporter tool to be used in mutant analysis. To transform this reporter into a tool that also permits the visualization of the shape of the expressing glial cell (and defects of glial shape in mutant backgrounds), we sought to separate GFP from H2B through the insertion of a stop codon. To this end, we designed a single guide RNA that directs Cas9 to cut a site 20 bp from the 3’ end of *gfp* ( Figure 1b ) *.* The cut is repaired with a ssODN repair template containing the stop codon. We found that the resulting animals display a substantial reduction in fluorescence, incomplete conversion of the signal from nuclear to cytoplasmic, and variability of the signal across individuals ( Figure 1c ). As an alternative strategy, we employed the T2A self-cleaving peptide, first used in *C. elegans* by Ahier and Jarriault (2014). We used the same guide RNA as used for the stop codon insertion, but a different ssODN repair template that contains the sequence of the T2A self-cleaving peptide (Ahier and Jarriault 2014) . This manipulation converts the SL2::GFP::H2B cassette into an SL2::GFP::T2A::H2B cassette and hence, during translation, the GFP should released from H2B, allowing it to diffuse throughout the cytoplasm and label the entire cell. In contrast to the stop codon insertion, we now observe a robust and consistent signal that fills the entire cytoplasm ( Figure 1d ). Residual GFP signal in the nucleus suggests that the cleavage by T2A is not 100% efficient. Our single guide RNA and repair template can be used for any locus tagged with a GFP::H2B reporter cassette.

## Methods

CRISPR/Cas9 genome engineering was performed as described by Dokshin *et al.* (2018). GFP reporters are from the class Fire vector kit (Fire *et al.* 1998). Out of 25 singled F1s that originated from injecting 15 adult P0 animals, eight plates had successful insertion of the STOP codon, as determined by PCR and subsequent sequencing. For insertion of the T2A sequence, the brightness of the signal under a fluorescence dissecting microscope enabled selection of animals in which the signal was converted from nuclear to cytoplasmic. For confocal microscopy, worms were immobilized with 50 mM sodium azide and mounted on glass slides with 5% agarose. Images were acquired with a Zeiss LSM 980 confocal microscope. ZEN software was used to generate maximum intensity projections of the images.

## Reagents

Guide RNA: 5’-TGCTGGGATTACACATGGCA-3’

T2A ssODN repair template: 5’-AGAGACCACATGGTCCTTCTTGAGTTTGTAACAGCTGCAGGCATCACTCACGGGATGGATGAACTATACAAAgagggcagaggaagtctgctaacatgcggtgacgtcgaggagaatcctggcccaCCACCAAAGCCATCTGCCAAGGGAGCCAAGAAGGC-3’

STOP ssODN repair template: 5’-AGAGACCACATGGTCCTTCTTGAGTTTGTAACAGCTGCAGGCATCACTCACGGGATGGATGAACTATACAAATAACCACCAAAGCCATCTGCCAAGGGAGCCAAGAAGGC

Strains:

PHX6670 : *spig-2 ( syb6670 [spig-2::sl2::gfp::h2b])*

OH18297 : spig-2 ( ot1324 [spig-2::sl2::gfp::t2a::h2b * syb6670 ])

OH19053 : spig-2 ( ot1469 [spig-2::sl2::gfp::stop::h2b * syb6670 ])
